# Dynamic Interactions between Induction and Reinforcement in the Organization of Behavior

**DOI:** 10.1007/s40614-025-00453-5

**Published:** 2025-06-03

**Authors:** Gabriela E. López-Tolsa, Ricardo Pellón

**Affiliations:** Animal Learning and Behavior Laboratory, Departamento de Psicología Básica I, Facultad de Psicología, Universidad Nacional de Educación a Distancia (UNED), C/Juan del Rosal 10, 28040 Madrid, Spain

**Keywords:** Behavioral dynamics, Schedule-induced behaviors, Reinforcement, Behavior organization

## Abstract

Behavior is dynamic because it results from the interactions between organisms and their environment. Reinforcement is the primary mechanism for explaining behavior, and it has evolved in various ways, allowing for the explanation of different aspects of behavior acquisition and maintenance. The adequacy of reinforcement in explaining behavior acquisition has mostly been tested on target behaviors. However, a broader understanding of behavior requires accounting not only for target behaviors but for all behaviors in a given situation. This article presents several experiments showcasing schedule-induced behaviors to analyze the variables that determine which behaviors are acquired and how they are organized. First, the effects of both physical and contingency-based constraints on the organization of behavior are examined. Second, the role of competition and collaboration between behaviors in determining their distribution is discussed. Third, a dual effect of reinforcers on behavioral patterns is proposed. It is concluded that behaviors interact with one another and with environmental stimuli, and behavioral patterns are continuously induced, updated, and reinforced. Data in this article highlight the need to focus on the moment-to-moment updating of behavioral patterns to fully understand behavioral dynamics.

Behavior is the result of the interactions between the organism and its environment, so it is, by definition, dynamic. Some changes in the environment—often referred to as reinforcers—are said to increase the probability of occurrence of a target behavior, most of the time overlooking their effects on other behaviors. This article challenges the traditional focus of behavior analysis, by emphasizing how reinforcers shape entire behavioral patterns, not just target responses.

The concept of reinforcement has its roots in Thorndike's (1911) Law of Effect, in which he proposed that when a behavior is followed by a *satisfying event*, the association between that behavior and the antecedent stimulus is strengthened. This idea was later refined by Skinner (1938), who proposed that reinforcers increase the probability of occurrence of (i.e., strengthen) the behavior that produced them. Although the general idea that behavior changes as a function of reinforcement remains, the concept of reinforcement itself has since evolved in several ways (see Cowie, 2020; Killeen & Jacobs, 2017a, 2017b; Shahan, 2017 for some deeper discussions on the term "strength").

Nowadays, it is assumed that reinforcers influence not only the immediately preceding target response, but also responses occurring earlier in time (Catania, 1971; Killeen, 2023). However, the effect of reinforcers on behaviors decreases exponentially as the delay between the response and the reinforcer increases (Killeen, 1994, 2023; Killeen & Pellón, 2013). Furthermore, proximity between a response and a reinforcer is sufficient for behavior to come under the control of reinforcement; thus, the term “contingency”—defined as a causal relationship between the response and the reinforcer—is not deemed necessary to explain behavior acquisition and maintenance (Killeen, 2023; Killeen & Pellón, 2013; Skinner, 1948; Thomas, 1981).

In addition, the effect of reinforcers does not appear to be only retrospective; evidence suggests that they also have a prospective influence, acting as discriminative stimuli of the conditions in which the upcoming reinforcer will be delivered (e.g., Cowie et al., 2011, 2017), thus favoring some behaviors over others (Baum, 2018; López-Tolsa et al., 2025a). Last, it has long been recognized that reinforcers do not act arbitrarily on any pattern of behavior; rather, the reinforcer must be relevant to the response (e.g., Domjan & Galef, 1983; Timberlake & Lucas, 1989), as not every reinforcer will automatically strengthen any given response (Breland & Breland, 1961).

The aim of this article is to further advance the concept of reinforcement by arguing that environmental constraints, behavioral interactions, and the dual action of reinforcers dynamically and continuously shape behavioral patterns comprised of target and other—nonarbitrary selected—behaviors. To illustrate this, we present data drawn from studies on schedule-induced behaviors.

## Schedule-Induced Behaviors

The study of schedule-induced behaviors offers an opportunity to deepen our understanding of the mechanisms underlying behavioral dynamics (López-Tolsa, 2019), as it allows for the examination of the sequential organization of behaviors beyond the target response. Schedule-induced behaviors are those that occur during interreinforcement intervals without an explicit contingency between their occurrence and the delivery of a reinforcer (Pellón et al., 2020). In other words, they are the other measurable (i.e., nontarget) behaviors that are relevant in an experimentally controlled situation.

The conditions required for the development of schedule-induced behavior are relatively simple: delivery of reinforcement under an intermittent schedule and having access to a stimulus that is phylogenetically relevant in the situation (e.g., schedule-induced drinking when the reinforcer is food, or schedule-induced running when the reinforcer is both food or water; see Lucas et al., 1988; Núñez-Santana et al., 2024; Timberlake & Lucas, 1985, 1991). The most studied example of schedule-induced behaviors is schedule-induced drinking (SID), which was first described by Falk (1961) as an outstanding (polydipsic) increase in water consumption during the experimental sessions when rats are exposed to an intermittent food schedule and they are deprived of food but not of water (Ardoy & Pellón, 2004; Castilla & Pellón, 2013; Flores & Pellón, 1995; Flory, 1971; Fuentes-Verdugo et al., 2023; Lamas & Pellón, 1997; López-Tolsa & Pellón, 2021; Pellón, 1992; Segal & Holloway, 1963).

Schedule induction is a general phenomenon that can be observed under a variety of reinforcement schedules (Pellón et al., 2020). However, the types of behaviors to be induced are highly dependent on the species’ behavioral repertoire, the type of reinforcer, and the constraints of the environment (see Breland & Breland, 1961; Silva & Timberlake, 1998). Schedule-induced drinking has been mostly studied in rats, but it has also been observed in monkeys (Helms et al., 2013), mice (Palfai et al., 1971) and human children (Gray Granger et al., 1984). Furthermore, other schedule-induced behaviors that have been observed include wheel-running in rats (Gutiérrez-Ferre & Pellón, 2019; Levitsky & Collier, 1968); head-movement (Buzzard & Hake, 1984), bolt-pecking (Miller & Gollub, 1974), and attack (Pitts & Malagodi, 1996) in pigeons; biting in squirrel monkeys (DeWeese, 1977); and pacing (Kachanoff et al., 1973), smoking (Wallace & Singer, 1976), and various behaviors (Wallace et al., 1975) in humans; among others. Moreover, traditional target behaviors—such as lever pressing in rats (Baum & Aparicio, 2020) and key pecking in pigeons (Mueller et al., 2023)—have also been reported to occur under similar circumstances.

Schedule-induced behaviors were first regarded as representing a different class of behavior that was an adjunct to the target behavior (i.e., adjunctive behaviors; Falk, 1971, 1977) and that was related to periods of low probability of reinforcement (Staddon, 1977). However, traditional operant behaviors and schedule-induced behaviors share several similarities that blur the boundaries between them (see Killeen & Pellón, 2013, for an in-depth characterization of schedule-induced behaviors as operants): (1) they are sensitive to changes in the frequency, quality, and quantity of the reinforcer (e.g., Castilla & Pellón, 2013; Lamas & Pellón, 1995; Núñez-Santana et al., 2024); (2) they can be developed in the absence of a contingency, yet are still subject to reinforcement and punishment (e.g., Álvarez et al., 2016; Pellón & Blackman, 1987); (3) schedule-induced behaviors are acquired at the same rate as operant behaviors maintained by delayed reinforcement (Killeen & Pellón, 2013); and (4) they exhibit a similarly organized bout-and-pause microstructure (Íbias et al., 2015).

The main difference between schedule-induced and operant behaviors is the location of each within the interreinforcement interval: conventional operant behaviors are mostly located at the end of the interreinforcement interval, whereas schedule-induced behaviors are mostly located at the beginning of the interreinforcement interval. However, as we will discuss later, in the lack of a target operant behavior, some schedule-induced behaviors usually occur at the end of the interval (Fuentes-Verdugo et al., 2023; Martínez‐Herrada et al., 2025), and explicit operant behaviors can occur at much earlier times than the reinforcer (e.g., Lattal & Gleeson, 1990). We argue that the main variable that will determine if a behavior develops into a traditional operant or an induced location is its proximity to the reinforcer. If a behavior is arbitrarily chosen as an operant, it normally occurs in close proximity to the reinforcers, but this does not mean that operants cannot be less proximal to reinforcers.

Moreover, some evidence suggests that the effect of reinforcers depends on where a specific response is located within the behavioral pattern and how specific the contingency is (López-Tolsa & Pellón, 2021; see also Honey et al., 2020, for a model capturing forward and backward associations in Pavlovian learning). For example, SID typically occurs at the beginning of the interfood interval, suggesting it may be more strongly controlled by the previous reinforcer inducing it (López-Tolsa et al., 2025a). On the other hand, schedule-induced magazine-entering, in the absence of a target behavior, may be equally controlled by the upcoming reinforcer as a (programmed) target behavior, with both exhibiting a similar distribution (scallop) and a comparable relationship to other behaviors occurring earlier in the interval (Fuentes-Verdugo et al., 2023; Martínez‐Herrada et al., 2025; Pellón et al., 2018).

In sum, the study of schedule-induced behavior provides an opportunity to understand the mechanisms that govern behaviors, including their selection, timing and organization. By examining which behaviors emerge and how they interact with each other and their environment, we can deepen our understanding of the broader dynamics of behavior beyond the simple contingencies established with target behaviors. The goal of this article is to analyze the variables that determine which behaviors are acquired and how they are organized. In particular, we focus on three interrelated factors: physical and contingency-based constraints of the environment, competition and collaboration between behaviors, and the dual (retrospective and prospective) effect of reinforcers on behavioral patterns.

## Constraints of the Environment

For the most part, behaviors occur sequentially, because not many behaviors are compatible with others, to be performed at the same time. This is particularly true in experimental contexts, in which not many operanda are available for manipulation, and the ones that are there, are often far enough that they cannot be easily manipulated at the same time (i.e., it is *possible* for a rat to press the lever with a paw and eat a food pellet at the same time, but it is not very easy, nor very common). Considering this, with enough training, the sequences of behaviors seem to be relatively stable as long as the same reinforcing conditions are maintained (López-Tolsa & Pellón, 2021; Martínez‐Herrada et al., 2025; Staddon & Simmelhag, 1971). Moreover, the order in which behaviors occur does not appear to be random, as certain regularities have been observed (Silva & Timberlake, 1998; Staddon, 1977; Staddon & Simmelhag, 1971).

In his seminal article on superstition, Skinner (1948) reported that pigeons developed seemingly idiosyncratic patterns of responses when they received food under a fixed time (FT) schedule. Skinner’s interpretation of his results was that the noncontingent delivery of food was reinforcing the occurrence of the random behavior that each bird happened to be doing in the moment of its delivery, thus naming it superstitious behavior. Later, Staddon and Simmelhag (1971) reported a replica showing that the patterns were not as idiosyncratic, nor random, as Skinner claimed, but that they were organized in specific ways that were common for all pigeons in the study. Based on their data, Staddon and Simmelhag (1971) proposed that organisms display a variety of behaviors phylogenetically appropriate to the situation, some of which (i.e., terminal responses) will be later selected by the reinforcer. Staddon (1977) further elaborated on that idea and distinguished three types of behaviors: interim, facultative, and terminal. Terminal behaviors are those that occur in close proximity to the upcoming reinforcer, like target or consummatory behaviors (e.g., lever-pressing, key-pecking, magazine-entering); interim behaviors are those that occur at the beginning of the interval (e.g., SID); and facultative behaviors would occur in between interim and terminal activities if time between reinforcers is long enough for their expression (e.g., wheel-running, self-grooming). This means that in a situation in which reinforcement is delivered intermittently and rats have a bottle of water, a running wheel, and a lever available, the expected pattern would be first spout-licking, then wheel-running, and finally lever-pressing, thus providing a phylogenetically predisposed behavioral pattern that would partially explain why SID occurs at the beginning of the interreinforcement interval (Staddon & Simmelhag, 1971).

Although the *phylogenetically predisposed* order of behaviors appears to be relatively fixed following preorganized patterns relevant to the situation (Staddon, 1977; Staddon & Simmelhag, 1971; see also Timberlake & Lucas, 1985), it serves as a foundation from which different behavioral patterns can occur through changes in the contingencies, allowing adaptation to environmental constraints (e.g., López-Crespo et al., 2004). Environmental constraints can be categorized into two types: physical constraints, which refer to the actual possibility of performing a behavior (e.g., a lever press cannot occur if there is no lever available), and contingency-based constraints, which relate to the flexibility or strictness of the reinforcement schedule.

Álvarez et al. (2011) conducted an experiment with the goal to evaluate if SID—the best-known example of interim behavior—could be developed at different moments in the interfood interval, depending on when water was available. In their experiment, rats were exposed to an FT 120-s schedule, so that a food pellet was delivered every 120 s regardless of the rats’ behavior. Thirty pellets were delivered in every session. In phase 1 water was available for Group I only during the first 30 s of the interval, corresponding to the period in which SID is usually located, whereas for Group II water was available only during the last 30 s of the interval, thus in the period in which the target or consummatory behavior, but not SID, usually develops. In Phase 2 water availability was reversed, so that Group I had access to water in the last 30 s of the interval, and Group II had access to water in the first 30 s of the interval.

**Fig. 1:**
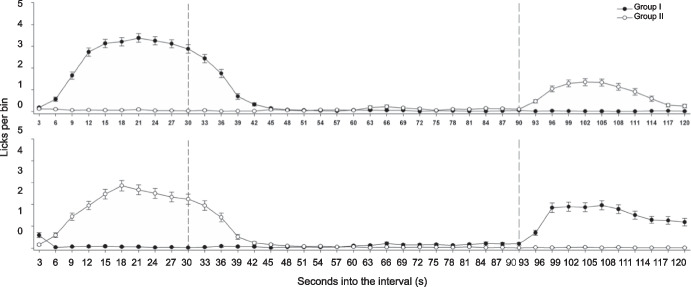
Distribution of Licking in 3-s Bins. *Note.* Upper panel is the last session of Phase 1, lower panel is the last session of Phase 2. Adapted from Álvarez et al. (2011)

Figure 1 shows that both groups of rats developed SID, although at a lower rate for Group II, likely because of competition with magazine entries occurring at the end of the interfood intervals. In both cases drinking showed the typical inverted u-shape distribution of licks along the interfood interval. When water availability changed, both groups drank in the corresponding time, swapping their distributions. These results show that although some behaviors might be phylogenetically predisposed to develop at a specific time, there is flexibility that allows them to occur at other times if the environment changes (see also,Gilbert, 1974; López-Crespo et al., 2004). When reinforcers are delivered periodically without explicitly reinforcing a specific behavior they will control behaviors occurring in proximity to them; thus, if a behavior can occur in proximity to the reinforcer, it can be reinforced.

Proximity has been proposed as the most fundamental mechanism by which events become connected (Killeen, 2023; Killeen & Pellón, 2013; Skinner, 1948), reducing the need for an imposed contingency to explain behavior acquisition. An alternative proposition is that contingency is an arrangement designed to constrain behavior so that it occurs at a specific rate or within a specific timeframe. When a contingency is strict (e.g., ratio schedules), it favors a more rigid behavioral pattern, whereas a more flexible contingency (e.g., time- or interval-based schedules) allows for greater variability in behavioral patterns. Proximity, however, does not require behavior and reinforcement to occur simultaneously but suggests that reinforcers have a stronger effect on behaviors that occur closer in time. The closer a behavior is to reinforcement, the greater the control the reinforcer exerts over it (Killeen, 2023; Killeen & Pellón, 2013). Although contingencies imply a causal relationship between behavior and reinforcement, proximity entails only a temporal relationship.

**Fig. 2:**
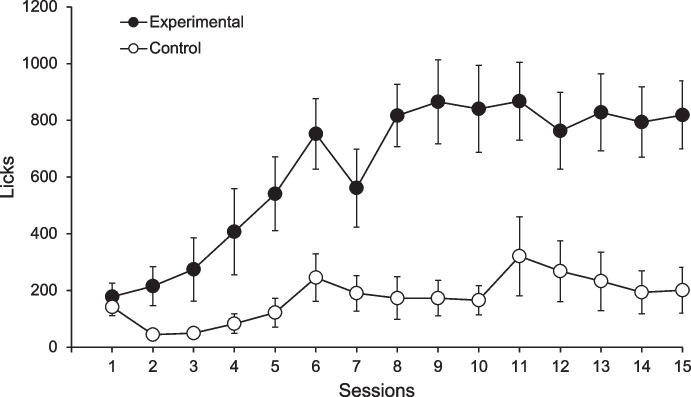
Total Licks per Session for the Experimental (Black Circles) and Control (White Circles) Groups. *Note.* Adapted from Álvarez et al. (2016)

**Fig. 3:**
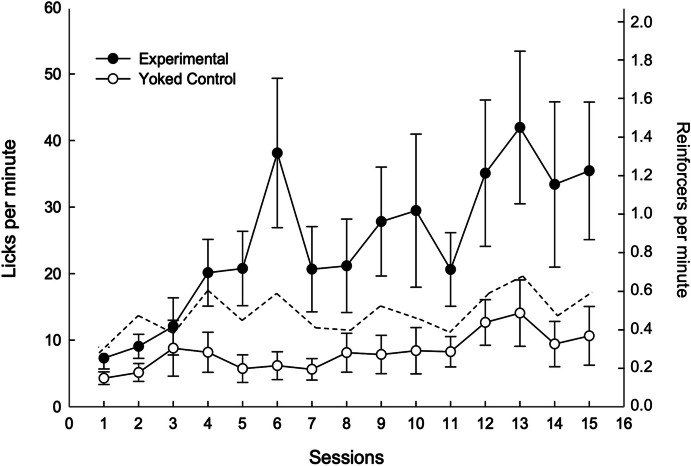
Licking Rate (Circles, Left y-axis) and Reinforcement Rate (Discontinued Line, Right y-axis) throughout the 15 Experimental Sessions. *Note.* Vertical bars are the SEM

Although contingencies often promote proximity between certain behaviors and the reinforcer, they can be understood as the computation of several contiguities (cf. Rescorla, 1967). Contingency ensures that events that occur separated in time stay connected, so the associations can be preserved even though the events are contiguous only when analyzed during extended periods. An example of this can be found in the work of Álvarez et al. (2016) where rats were initially exposed to an FT 90-s schedule for which a single pellet of food was delivered every 90 s. Experimental rats could accelerate food delivery by licking the spout: if they licked at least 30 times, food was dispensed after 30 s instead of 90 s (reducing the interfood interval by 20 s for every 10 licks). Yoked control rats received food at the same time as their paired experimental rat but had no control over the rate of food delivery. Figure 2 shows that experimental rats developed four times more licking behavior than their controls, although licking occurred at the beginning of the interfood interval in both groups (see Fig. 3 in Álvarez et al., 2016). This indicates that, despite the long delay between licking and food delivery, licking rates were still controlled by the contingency between its occurrence and reinforcement.

In a similar unpublished^1^ experiment, food pellets were delivered according to a tandem FR 20 FT 30-s schedule, so that the reinforcer was contingent on the performance of 20 licks, but with a 30-s delay between the 20th lick and the food. Licking could also occur during the FT component, but no additional contingency operated during this time. This contingency constrained licking to occur at the beginning of interfood intervals, because it is characteristic of schedule-induced behavior (Pellón et al., 2020). Control rats were yoked to the experimental ones, receiving the food pellets at the same time, but without a response-reinforcer contingency between their licks and the reinforcer.

Figure 3 shows the mean licking rate for the experimental and control groups during the 15 experimental sessions of training. All rats started with low licking rates during the first three sessions, but from the fourth session onwards experimental rats (black circles) began to drink more than control rats (white circles). By the end of training, animals in the experimental group showed a significantly higher rate of about 40 licks per minute on average, whereas control rats showed a rate of 10 licks per minute (main effect of Group: *F*_(1,14)_ = 15.14, *p* = 0.002; main effect of Session:* F*_(4.63,64.75)_ = 12.93, *p* < 0.001; and Session x Group interaction: *F*_(4.63,64.75)_ = 5.14, *p* < 0.001).^2^ The control group, in fact, hardly developed SID. Licking rate was approximately four times higher in experimental than in control rats, similar to what Álvarez et al. (2016) reported (see Fig. 2); also, licking was located at the beginning of interfood intervals, separated by a relatively long delay from food delivery.

Figure 3 also shows the mean reinforcement rate (discontinued line) for all rats across experimental sessions (noting that experimental and control rats received food at exactly the same time). Visual inspection of the data indicated that the reinforcement rate appeared to increase as training progressed, rising from 0.3 to nearly 0.6 reinforcers per minute between the first and last experimental sessions. This provides evidence that the experimental animals learned the lick–food contingency. The final rate corresponds to an average of approximately one food pellet every 2 min, which explains the low response rate of control rats at the end of training (similar again to Álvarez et al., 2016).

On the other hand, experimental rats exhibited the typically high rates of schedule-induced drinking despite the low reinforcement rate and long delays between licking and reinforcement, due to the added contingency between licking and food delivery. Moreover, the increase in licking rates among control rats over the course of the experiment appeared to track the gradual increase in reinforcement rate, as inferred from visual inspection of both trends. However, the additional increase in licking rates among experimental rats must be attributed to the contingency that required licking for food pellets to be delivered. Studies on reinforcement independent of behavior have shown that response rates decline compared to procedures where reinforcement is response-dependent (e.g., Zeiler, 1968), similar to the findings reported here.

These results also go in line with the finding that operant responses can be acquired and maintained with long delays between them and the reinforcer (e.g., Lattal & Gleeson, 1990). Notwithstanding, the degree of acquisition depends on the length of the delay between the response and the reinforcer, something that has been observed both with traditional operant (Keely et al., 2007) and induced behaviors (Lamas & Pellón, 1995). Furthermore, it has been shown that the responding rate depends on the length of the response-reinforcer delay (see Pellón et al., 2018; Pellón & Pérez-Padilla, 2013).

As observed above, the establishment of a contingency that favors a delay between the licks and the delivery of the reinforcer will determine that a reinforced behavior occurs at the beginning of the interval. In that sense, the degree of flexibility of a contingency can determine the location of a behavior, but also the order in which behaviors occur in the interreinforcement interval. The order and consistency of patterns developed by different subjects may vary depending on the degree of flexibility in the contingency. Ratio schedules will favor the development of patterns that only (or mostly) include the target behavior, whereas schedules with more flexible contingencies like time or interval ones, will favor patterns that end with the target response, regardless of the responses that precede it, so they may vary significantly among subjects (Killeen, 2023).

If a flexible and a strict contingency are combined within the same schedule, different subjects will likely develop different response patterns. In another unpublished experiment, licking was reinforced with food under a conjunctive FT 60-s FR 10 schedule, meaning reinforcement was delivered only after both criteria were met: the rats had to complete at least 10 licks, and 60 s had to elapse since the previous reinforcer. If the rats completed 10 licks before the 60 s elapsed, the food pellet was delivered precisely at 60 s. If 60 s had elapsed but the rats had not completed 10 licks, the food pellet was delivered immediately after the 10th lick. Sessions continued until 60 reinforcers were delivered (typically lasting close to 60 min).

**Fig. 4:**
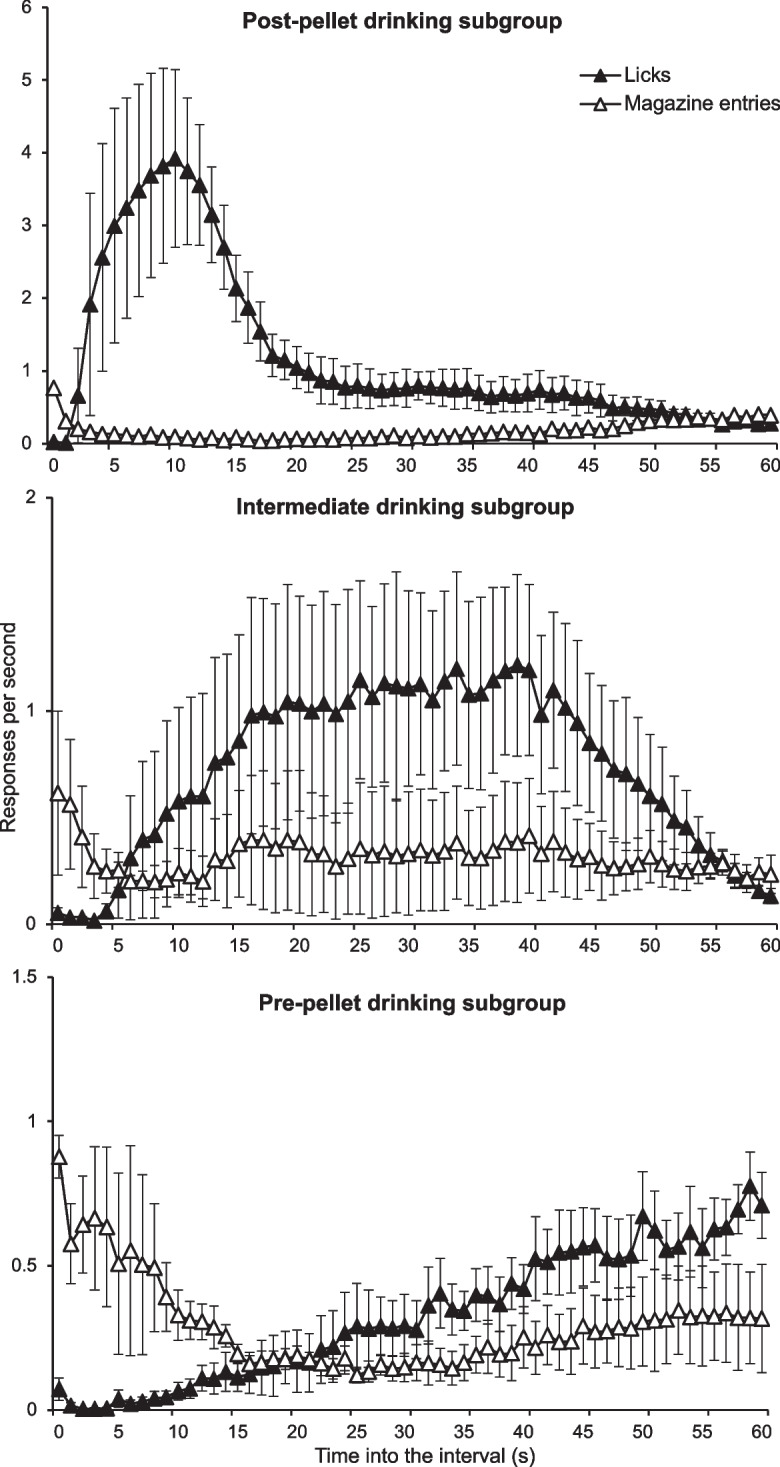
Distribution of Licking (Black Triangles) and Magazine-Entering (White Triangles) in 1-s Bins. *Note.* Vertical bars are the SEM

Figure 4 shows that three distinct response patterns emerged under this schedule: some rats developed the typical post-pellet drinking (upper panel, *n* = 3); others drank primarily in the middle of the interval (middle panel, *n* = 3); and a third group exhibited pre-pellet drinking (lower panel, *n* = 4). Although all subjects in these groups received food at the same rate, the allocation and sequence of behaviors varied. Such variations in behavioral order are more common under schedules with flexible contingencies (i.e., time- and interval-based schedules) and likely originate in the early stages of training (Killeen, 2023).

The reinforcer acts upon behaviors preceding its delivery (Catania, 1971), reinforcing behavioral patterns rather than discrete responses (López-Tolsa & Pellón, 2021). If a behavior occurs at the end of an interval and does not preclude or delay reinforcer delivery (i.e.*,* it does not compete with the target response), the reinforcer will increase the probability of that specific pattern occurring in that particular sequence. Moreover, if there is a delay between the target response and reinforcer delivery, a pattern that begins with the target response but ends with a different one will also be reinforced—similar to what was observed with the post-pellet group in this experiment (see also Grosch & Neuringer, 1981; Mueller & Zentall, 2021; Ramos et al., 2019). Reinforcers strengthen whichever behaviors occur in close temporal proximity to them, particularly when the contingency is flexible enough to allow for a variety of behaviors to emerge at that time.

In addition, for a behavior to come under the control of reinforcement, it may first need to occur in close proximity to the reinforcer. However, due to competition with other behaviors, it may later shift to a different part of the interval. In line with this, we conducted an unpublished experiment to examine how the number of pairings per session affects the development of SID by altering the probability that drinking occurs immediately before or in close temporal proximity to food delivery. We hypothesized that SID must occur in proximity to the reinforcer for several trials before it can be reinforced by food delivery, a process that is more likely to occur during longer sessions (60 trials) than during shorter ones (30 trials).

**Fig. 5:**
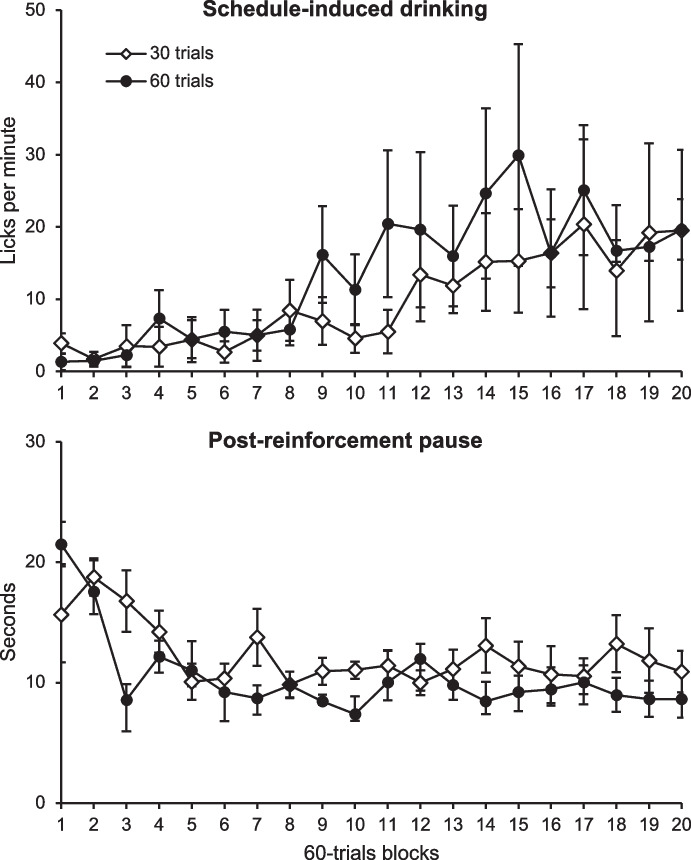
Licking Rate (Upper Panel) and Postreinforcement Pause (Lower Panel) throughout the Experiment in 60-Trial Blocks. *Note.* Vertical bars are the SEM

Two groups of rats were exposed to an FT 30-s food schedule, in which a single food pellet was delivered every 30 s regardless of the rats’ behavior. One group received 60 trials per daily session, whereas the other received 30. The experiment lasted for 1,200 trials for both groups (20 sessions of 60 trials and 40 sessions of 30 trials, respectively). The top panel of Fig. 5 shows the licking rate in 60-trials blocks, indicating that SID reached higher levels in the 60-trials group by the midpoint of the experiment. However, mean licking levels became similar between the 60- and 30-trial groups toward the end, reflecting the main effect of the overall number of potential pairings between licking and the reinforcer. Moreover, although an ANOVA^3^ reveled there were no significant differences between groups, nor group by sessions interaction effect, only two of the five rats in the 30-trial group developed SID, whereas four of the five rats in the 60-trial group did (minimum criterion = 10 licks/min).

The bottom panel of Fig. 5 shows the median postreinforcement pause (PRP) for the first lick in the two groups of rats (i.e., the time between food delivery and the first lick). At first, the PRP was longer in the 60-trial group (22 s) than in the 30-trial group (16 s) during the first block. However, it rapidly declined as the trial blocks progressed, eventually stabilizing at approximately 10 s after food delivery—a period during which the food was also collected and consumed. An ANOVA^4^ revealed there were no significant differences between groups, nor interaction group by sessions.

These results indicate that the number of pairings between a behavior and the reinforcers is relevant for the behavior to increase significantly, which in this experiment occurred at about the 9th block for the 60-trial group and a few blocks later for the 30-trial group, thus implying that massive training might favor the development of SID, although not enough to accelerate acquisition significantly, as in the present experiment. In line with the hypothesis stated above, the longer PRP at the beginning of the experiment suggest that SID initially occurs in proximity to the reinforcer, where it is more likely to be reinforced, and is later displaced due to competition with terminal behaviors.

## Competition and Collaboration

Competition has often been considered a central concept to understand the dynamics of behaviors, because for one behavior to increase in rate or duration, other(s) have to decrease (Baum, 2018; Killeen, 2023; Pellón & Killeen, 2015). A less explored process is the collaboration between behaviors, as they, together with environmental constraints, shape each other’s distribution (Pellón & Killeen, 2015). For example, López-Tolsa and Pellón (2021, Fig. 2) demonstrated that, under FI schedules, the point at which SID ends within the interreinforcement interval determines the onset of lever-pressing. In particular, the onset of lever-pressing was delayed under shorter schedules (e.g., FI 15 s and FI 30 s) and advanced under longer ones (e.g., FI 60 s) compared to rats that did not develop SID. Furthermore, when behavioral patterns are long enough, it might be assumed that they are maintained in the same way as chained behaviors (Thrailkill & Bouton, 2016), so that the behavior closer to the upcoming reinforcer is actually reinforcing the behaviors preceding it (Killeen, 2023).

Martínez-Herrada et al. (2025) conducted an experiment to examine the development and distribution of SID and magazine entries when either a highly valued or a less valued flavor of food pellets was delivered. Rats were divided into two groups: one received food pellets with their preferred flavor, whereas the other received their least-preferred flavor. Subjects were exposed to three FT schedules (30, 60, and 120 s) in different phases of the study, with pellets delivered at fixed intervals regardless of the rats' behavior.

**Fig. 6:**
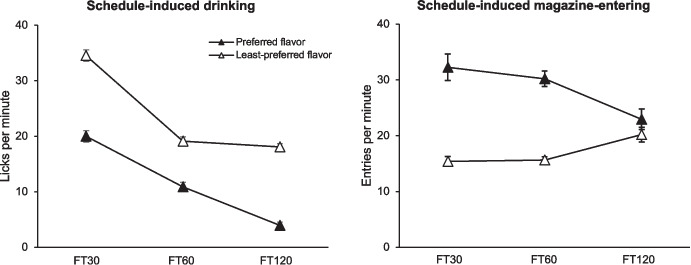
Mean Response Rate in the Last Three Sessions of Each FT Schedule. *Note.* Left panel depicts licking rate and right panel depicts magazine-entering rate. Adapted from Martínez-Herrada et al. (2025)

Figure 6 shows the mean response rate from the last three sessions of each FT schedule. Magazine entries (right-hand panel) were higher for the preferred-flavor group (black symbols), whereas SID (left-hand panel) was higher for the least-preferred flavor group (white symbols). These opposing trends align with the principle of competition: when one behavior increases, the other must decrease. However, during FT 120-s, differences in magazine entries largely disappeared, whereas differences in drinking remained similar to those observed in FT 30 s (and smaller than in FT 60 s).

SID typically occurs at higher rates under FT 30 and 60 s compared to FT 120 s (Pellón et al., 2020), because it tends to locate early in the interval, which results in longer delays between the last lick and food delivery, reducing the likelihood of drinking being initially reinforced. However, in the experiment by Martínez-Herrada et al. (2025), the transition time (when magazine-entering rates exceeded licking rates) was later for the least-preferred group (49.70 s) than for the preferred group (38.02 s), effectively maintaining a shorter delay between drinking and reinforcement. The least-preferred food induced fewer magazine entries—the behavior occurring closest to reinforcement—allowing SID to develop at higher rates and persist later in the interval (see Fig. 3 in Martínez-Herrada et al., 2025). These results show that the time allocated to each behavior is important, but the sequence in which behaviors occur is equally crucial, as preceding behaviors seem to signal the onset of subsequent ones.

Ardoy and Pellón (2004) examined the interaction of behaviors by removing one of them, and found evidence that interactions between behaviors not only initiate subsequent behaviors in a chain but can also reinforce preceding ones. They exposed rats to an FI 60-s food-reinforcement schedule for lever-pressing with access to water in the experimental chamber. Rats developed lever-pressing and SID, and when performance was stable, they were divided into two groups and submitted to two successive test sessions. During test sessions, food was dispensed according to an FT 60-s schedule, regardless of the subjects’ behavior. For rats in the no-lever (experimental) group, the lever was retracted, whereas for rats in the lever (control) group, the lever was present but inactive.

**Fig. 7:**
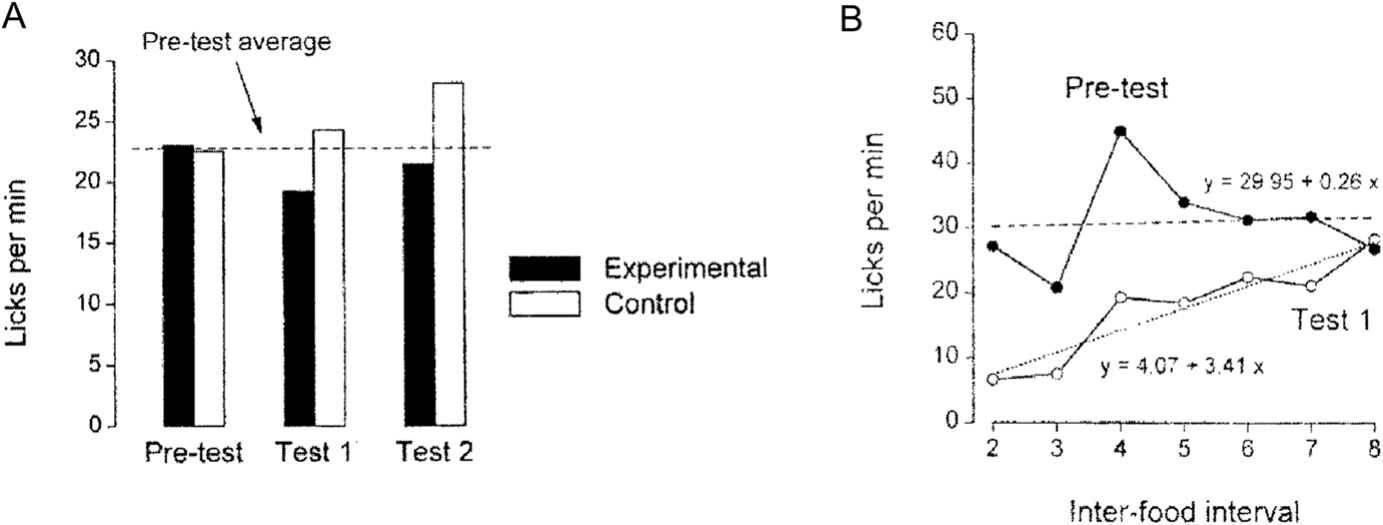
Note. A: Mean licking rate for the no-lever (experimental, black symbols) and lever (control, white symbols) groups during the last session of training, and the two test sessions. B: intra-session changes in the mean licking rate in intervals 2 to 8, for the experimental group. Black symbols represent the last session of training and white symbols the first test session. Lines represent the best linear fit for each session. Figures 2 (panel **A**) and 4 (panel **B**), reprinted with permission from Ardoy and Pellón (2004)

Drinking declined for the no-lever rats, but not for the lever rats, although gross differences were not significant (Ardoy & Pellón, 2004) because the effect proved to be transitory, as can be seen in both panels of Fig. 7. The panel to the left depicts overall licks per minute during the final pretest session and the two test sessions, showing that drinking declined for the experimental rats, whereas for the control rats a slight increase in the rate of licking was observed. The panel to the right shows the rate of licking in the first eight interfood intervals of the first test session for the no-lever rats, showing that licks per minute first decreased, and then slowly recovered to rates similar to the pretest session, evidencing the transitory duration of the effect.

Results in Fig. 7 show that removing the opportunity to perform a behavior (lever-pressing) did not lead to an increase in the other potentially competing response (spout-licking). Instead, the interactions between these behaviors appeared to follow additional rules. Ardoy and Pellón (2004) had suggested that the animals may have learned a heterogeneous behavioral chain, first licking and then lever pressing, and that removing the second element would result in a temporary decrease in the preceding behavior, as was observed. This pattern is consistent with findings from explicitly trained chained behaviors (Thrailkill & Bouton, 2016).

This basic finding has been replicated several times in our laboratory and has shown that the order of behaviors in the chain is a relevant factor in the effect caused by the removal of a behavior (López‐Tolsa et al., 2025). Complementary findings have been reported by Boakes et al. (2015), who showed that the rate of magazine entries increased when the opportunity to drink water was available compared to when it was not, under an FT food delivery schedule. Similar event interactions have also been observed in Pavlovian conditioning (Urcelay, 2017). Moreover, this effect is likely dependent on the length of the interreinforcement interval, with competition being more pronounced at shorter intervals (López-Tolsa & Pellón, 2021). In addition, the transient nature of this phenomenon highlights the continuous adaptation of behavior, as demonstrated in other studies (e.g., López‐Tolsa et al., 2025).

Killeen and Pellón (2013) suggested that all behaviors occurring within interreinforcement intervals are reinforced simultaneously by food, though not to the same extent. Magazine entries and lever presses are typically reinforced with shorter delays, because they occur later in the interval, and are therefore expected to increase more rapidly than behaviors occurring earlier in the interval (e.g., see Ardoy & Pellón, 2004, for lever-pressing; and Boakes et al., 2015, for magazine-entering). As target and consummatory behaviors increase with continued training, they tend to displace other behaviors, such as licking, to earlier parts of the interval. This is supported by observations showing that licking shifts backward as training progresses (as seen in the last experiment discussed in the previous section; see also Patterson & Boakes, 2012; Staddon & Ayres, 1975). These findings support the hypothesis that licking is initially acquired in close proximity to food delivery and that, over time, and due to competition, the delay between licking and reinforcement gradually increases (see also Álvarez et al., 2016).

The data discussed here are consistent with the hypothesis that behaviors are organized into chained patterns. First, reinforcers increase the rate of related behaviors (Killeen, 2023; Killeen et al., 1978). Then, depending on environmental constraints (López-Tolsa & Pellón, 2021), and through competition and collaboration (Ardoy & Pellón, 2004; Martínez-Herrada et al., 2023; Pellón & Killeen, 2015), stable patterns emerge in which behaviors depend on one another while also interacting with the reinforcer. The dual effect of reinforcers on stable behavioral patterns is discussed next.

## Dual Effect of Reinforcers on Behavioral Patterns

Reinforcers do not only strengthen the behavior that immediately precedes them, but they seem to have an almost immediate effect on the behavioral pattern being performed in a given situation. The delivery of a reinforcer, as well as other environmental changes (Morse & Skinner, 1957; Sheehan et al., 2012; Starr & Staddon, 1982), typically have a dual effect on behavioral patterns: they interrupt an ongoing pattern and subsequently reinitiate it (López-Tolsa & Pellón, 2021). Two experiments illustrating this effect are described next.

A group of rats was exposed to 30 sessions of an FI 15-s food-reinforcement schedule with lever-pressing as target behavior and free access to water. At the end of this experimental phase, rats showed the expected distribution: rats licked during most of the interval and then started lever-pressing at higher rates at the end of the interval (see Fig. 3, panel A, in López-Tolsa & Pellón, 2021). From the 31st session onwards, a peak procedure (Catania, 1970; Church et al., 1991; Roberts, 1981) was implemented to evaluate timing, so six peak interval (PI) trials (three times longer the FI trials, thus 45 s) were randomly intercalated in each 30 trials session. The peak procedure lasted for 30 sessions.

**Fig. 8:**
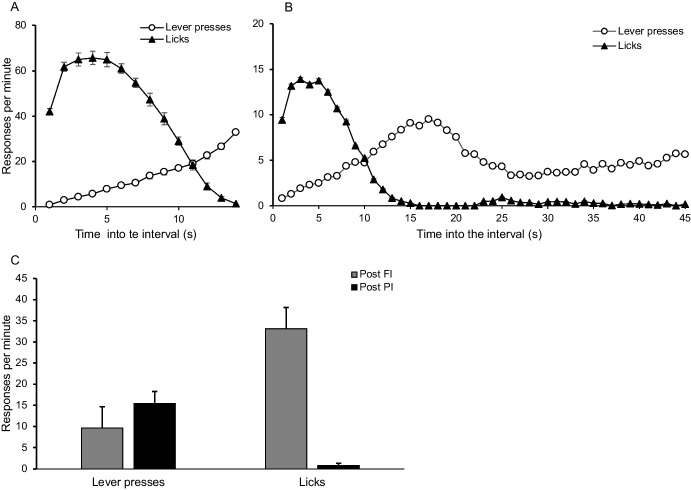
Note. Panel A: distribution of responses during fixed interval trials. Panel B: distribution of responses during peak interval trials. Panel C: Response rate in the post-FI and post-PI trials. White circles represent lever-presses and black triangles represent licks. Vertical bars are the SEM

Data from the last five sessions of the peak procedure are presented in Fig. 8. The distribution of responses during the regular FI trials remained the same as in the previous phase (panel A). In contrast, the distribution of responses during the PI trials (panel B) showed drinking at the beginning of the interval, followed by an increasing rate of lever-pressing that peaked at second 15, and then a decline to about half the peak response rate until the end of the trial. Isolated and nonsystematic bouts of licking were occasionally observed following the decrease in lever-pressing, as indicated by slight increases in licking from second 23 onward. It was initially hypothesized that SID would reappear after the decrease in lever-pressing due to competition; however, this did not occur. Furthermore, a deeper analysis revealed that SID only occurred after the delivery of a reinforcer.

Panel C in Fig. 8 shows the response rate of licks and lever-presses in the trials that followed the delivery of a reinforcer (post-FI trials) and the response rate of licks and lever-presses that followed the absence of a reinforcer (post-PI trials). Licking rate was significantly^5^ higher during post-FI trials than during post-PI trials (*t*_(6)_ = −4.75, *p* = 0.003), whereas lever-pressing rate showed the opposite trend (*t*_(6)_ = 2.72, *p* = 0.034). This result suggests that lever presses (i.e., the last behavior in the pattern) continued until it was “interrupted” by the delivery of a reinforcer, and that the behavioral pattern was only restarted after the delivery of a reinforcer, thus highlighting the importance of direct interactions between behaviors and reinforcers.

If we were to ignore the distribution of responses, we would observe that reinforcement rate decreases during post-PI trials because no reinforcer is delivered, whereas the lever-pressing rate increases compared to post-FI trials. It might be true that licking in post-PI trials decreases due to competition with lever-pressing; however, changes in response rate depend on the occurrence (or lack of) of a specific event (reinforcer), thus highlighting the need to analyze specific local interactions between behaviors and reinforcers. Reinforcers do not necessarily always immediately induce the target behavior (Catania, 1971; Cowie et al., 2017), but they do stop the behavior that is being performed in that moment and induce the first behavior included in the previously learned pattern.

An experiment that better illustrates how reinforcers interrupt an ongoing behavioral pattern was conducted using a bisection-task procedure (Church & Deluty, 1977). During the training phase, rats were presented with a light that lasted either 10 or 40 s. If the light lasted 10 s, pressing the right lever resulted in a food pellet, whereas if the light lasted 40 s, pressing the left lever led to reinforcement (lever assignments were counterbalanced across animals). Each trial proceeded as follows: the light was illuminated for the designated duration (10 or 40 s), after which it was turned off and the levers were inserted into the chamber. Upon the first lever press, the levers were retracted. If the rat pressed the correct lever, a food pellet was delivered, followed by a 3 s intertrial interval (ITI). If the rat pressed the incorrect lever, no pellet was delivered, and the 3-s ITI began. Rats successfully learned to discriminate between the two light durations after an average of 27 sessions, at which point they moved to the test phase.

**Fig. 9:**
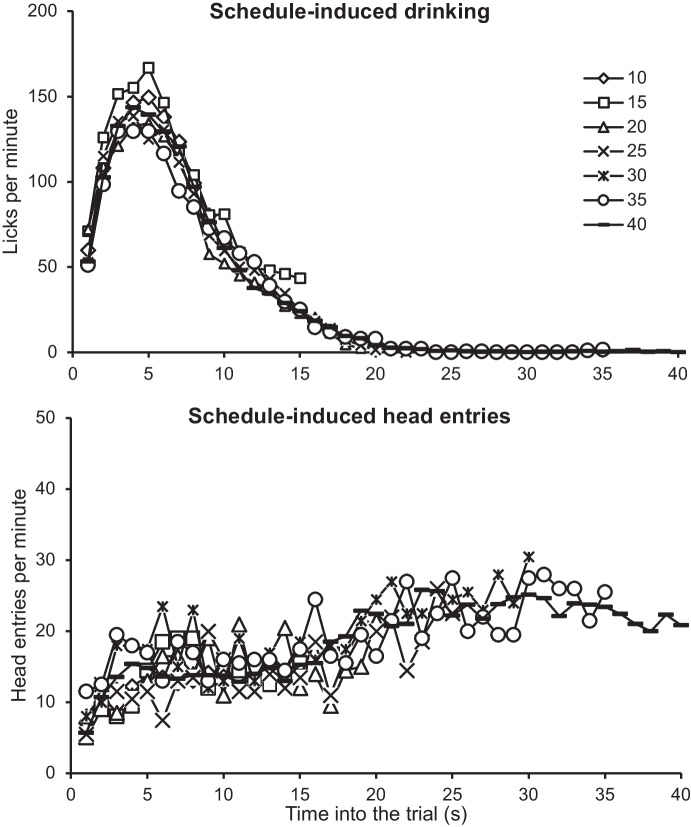
Distribution of Licks (Upper Panel) and Magazine-Entries (Lower Panel) throughout the Duration of the Stimulus. *Note.* Notice stimuli had different durations, so the curve is cut when the stimulus ended

During testing, in addition to the original training stimuli, five intermediate light durations (15, 20, 25, 30, and 35 s) were introduced. Throughout the experiment, rats had access to water in the chamber and developed a consistent behavioral pattern: they drank water during the first 20 s of each trial and then engaged in magazine-entering for the remaining time until the levers were inserted, and a food pellet was either delivered or omitted (Fig. 9). This pattern has been observed in previous studies (Fuentes-Verdugo et al., 2023; Martínez et al., 2025). It should be noted that the same pattern was maintained regardless of trial duration, indicating that lever insertion interrupted the ongoing behavioral sequence, whereas the start of the next trial reinstated it. Similar findings have been reported in other choice studies (Cleaveland et al., 2003; Grosch & Neuringer, 1981; Machado & Keen, 1999; Mueller & Zentall, 2021), suggesting that choice may not always involve selecting one response over another but rather engaging in a behavioral pattern that leads to a specific response occurring at a specific time (López-Tolsa & Pellón, 2021).

## Concluding Comments

Although the importance of "other" behaviors has been recognized for a long time (e.g., Falk, 1961; Skinner, 1948), the experimental analysis of behavior has mostly focused on studying the variables that directly affect target behaviors. However, for a complete understanding of the basic mechanisms responsible for behavior acquisition and maintenance, attention must also be directed to other behaviors in the situation, because their interaction with the target behavior can influence the rate and distribution of target behaviors.

One reason for studying only the target behavior is that measuring and controlling other behaviors is not always feasible, because it could reduce experimental control. However, measuring multiple behaviors in a situation, especially when they interact, would enhance external validity and provide better avenues for translatability. The study of schedule-induced behaviors offers a balance between experimental control and the measurement of multiple behaviors. Schedule induction is a well-studied and reliable phenomenon (Pellón et al., 2020), and schedule-induced behaviors are sensitive to most of the same variables as target behaviors, thus allowing for reliable results (Álvarez et al., 2016; Killeen & Pellón, 2013; Pellón & Blackman, 1987).

The data in this article outline three important variables in the development of behaviors. In a more flexible environment, behaviors tend to follow a phylogenetically predisposed order (Staddon, 1977; Staddon & Simmelhag, 1971); however, all behaviors are highly sensitive to physical and contingency-based constraints that may alter this predisposed order (Álvarez et al., 2011, 2016). Likewise, behaviors interact with each other through competition and collaboration: as one behavior increases, others must decrease, but the occurrence of one behavior can function as a discriminative stimulus or a reinforcer for other behaviors (Ardoy & Pellón, 2004; Pellón & Killeen, 2015). Last, reinforcers have a dual effect on behavioral patterns, as the delivery of a reinforcer tends to both stop the ongoing behavior and induce the beginning of a new behavioral pattern (López-Tolsa & Pellón, 2021).

In conclusion, behaviors interact with each other and with stimuli in the environment, and behavioral patterns are continuously induced, updated, and reinforced. A recent trend emphasizes time spent on a behavior and the covariance between behavior and reinforcer as primary variables for understanding behavior (Baum, 2018). However, the data in this article suggest that focusing on local interactions between behaviors, analyzing the order in which they occur, and examining the moment-to-moment updating of behavioral patterns can provide a more comprehensive understanding of behavioral dynamics.

## Data Availability

A repository was not set up for the article because it includes data from different projects, but data are available upon request.

## Footnotes

1 A general method for unpublished experiments is provided as supplementary material.

2 A mixed-design ANOVA was conducted to examine the effect of Group (experimental, control) and Session (15 sessions) on the number of licks per session. The degrees of freedom were corrected using the Greenhouse–Geisser estimate of sphericity, because Mauchly’s test indicated that the assumption of sphericity was violated for the main effect of Session and for the Session x Group interaction (*p* < 0.5). All analyses were conducted using JASP (JASP Team, 2024).

3 A mixed-design ANOVA was conducted to evaluate the effects of Group (30-trials vs. 60-trials) and Session (20 levels) on licking rate. There was a significant effect of Session (*F*_(19, 152)_ = 4.23, *p* < .001), but no significant effects of the Session x Group interaction (*F*_(19, 152)_ = 0.58, *p* = .916), nor a main effect of Group (*F*_(1, 8)_ = 0.33, *p* = .583).

4 A mixed-design ANOVA was conducted to examine the effect of Group (30-trial vs. 60-trial) and Session (20 sessions) on the PRP. There was a significant effect of Session (*F*_(19, 133)_ = 4.41, *p* < .001), but no significant effect of the interaction Group x Session (*F*_(19, 133)_ = 1.57, *p* = .073), nor was there an effect of the Group (*F*_(1, 7)_ = 0.94, *p* = .366).

5 A paired-sample *t*-test was conducted to assess the difference between response rates following peak intervals (post-PI) and fixed intervals (post-FI). Prior to the analysis, the assumption of normality was evaluated using the Shapiro–Wilk test. Neither lever-pressing nor licking rates deviated significantly from normality.
